# Metabolomics reveals changes in soil metabolic profiles during vegetation succession in karst area

**DOI:** 10.3389/fmicb.2024.1337672

**Published:** 2024-06-26

**Authors:** Chaofang Zhong, Cong Hu, Chaohao Xu, Zhonghua Zhang, Gang Hu

**Affiliations:** Key Laboratory of Wildlife Evolution and Conservation in Mountain Ecosystem of Guangxi, College of Environmental and Life Sciences, Nanning Normal University, Nanning, China

**Keywords:** karst, soil metabolomics, vegetation succession, metabolic network, maltotetraose

## Abstract

Soil metabolites are critical in regulating the dynamics of ecosystem structure and function, particularly in fragile karst ecosystems. Clarification of response of soil metabolism to vegetation succession in karst areas will contribute to the overall understanding and management of karst soils. Here, we investigated the metabolite characteristics of karst soils with different vegetation stages (grassland, brushwood, secondary forest and primary forest) based on untargeted metabolomics. We confirmed that the abundance and composition of soil metabolites altered with vegetation succession. Of the 403 metabolites we found, 157 had significantly varied expression levels across vegetation soils, including mainly lipids and lipid-like molecules, phenylpropanoids and polyketides, organic acids and derivatives. Certain soil metabolites, such as maltotetraose and bifurcose, were sensitive to vegetation succession, increasing significantly from grassland to brushwood and then decreasing dramatically in secondary and primary forests, making them possible indicators of karst vegetation succession. In addition, soil metabolic pathways, such as galactose metabolism and biosynthesis of unsaturated fatty acids, also changed with vegetation succession. This study characterized the soil metabolic profile in different vegetation stages during karst secondary succession, which would provide new insights for the management of karst soils.

## Introduction

The karst ecosystem is one of the most ecologically fragile ecosystems in the world, accounting for ∼12% of the Earth’s land surface area and being extremely vulnerable to soil erosion and rock desertification (Kaufmann, 2014; Sullivan et al., 2014). The natural restoration of karst’s rock desertification follows the law of secondary succession (grassland-brushwood-secondary forest-primary forest) (Qi et al., 2013). Karst vegetation succession is an ecological phenomenon involving predictable changes in plant communities, which can be used as indicators of environmental change (Pregitzer and Euskirchen, 2004). Karst vegetation succession influences both aboveground (e.g., plant communities) and belowground (e.g., soil bacterial communities) components (Francioli et al., 2018). Some studies have reported changes in soil function and metabolism along karst vegetation succession (Liu et al., 2015; Zhang et al., 2021). Plant communities at different successional stages produce different metabolites that alter soil ecology and respond to environmental demands (Zhang et al., 2015). Thus, a better understanding of the effects of vegetation succession on soil metabolism is critical, particularly in karst areas where ecosystems are fragile and prone to vegetation and soil degradation.

Soil metabolites are the molecular substances produced by the metabolism of plants, microorganisms, etc., which have an important effects on plant growth and soil ecosystem function (Pétriacq et al., 2017). The types and abundance of soil metabolites are influenced by soil microorganisms, plants and environmental factors, which may reflect soil quality, soil biodiversity and metabolic activities (Kudjordjie et al., 2019; Liu et al., 2020). Soil metabolomics is an emerging technique in recent years to reflect the metabolic characteristics of soils (Jones et al., 2014; Swenson et al., 2015). The technique allows the identification of biochemical intermediates in interacting metabolic pathways, leading to a better understanding of soil biology and ecology. It offers the possibility of improving the understanding of soil metabolism in a variety of natural environment ecosystems, especially karst ecosystems.

The metabolite response during vegetation succession is a reflection of the adaptation of plant communities and microbial communities to environmental change. In recent years, knowledge of the soil metabolome in karst ecosystems has increased, and attention has turned to its significance in soil functional diversity. Recent research has demonstrated that karst rocky desertification causes changes in root metabolites, which alter the survival and activities of microorganisms (Tang et al., 2024). Typical karst soil bacterial metabolomes are involved in the dissolution and formation of karst minerals, promote soil nutrient cycling, and contribute significantly to soil fertility and plant succession (Lian et al., 2011; Yuan et al., 2011). While attention has been paid to the soil properties and microbiomes at different successional stages of karst (Zhu et al., 2012), the microbial functions, such as their metabolism, are not available through soil properties and microbiome (Jansson and Hofmockel, 2018), the soil metabolome resources in karst ecosystems are mainly untapped. In addition, the influence of different vegetation types on soil metabolism should be important (Yan et al., 2022), but there is a lack of reports in this regard in karst areas. The vast unknowns of the karst ecosystem are increasing interest in the soil metabolome, and it is necessary to characterize soil metabolism at different successional stages to monitor changes in soil metabolic quality with vegetation. Understanding karst soil metabolites provides insights into how to support healthy karst soil ecosystems and sustainable agricultural practices.

To advance our understanding of the effects of secondary succession of karst vegetation on soil metabolites, this study used untargeted metabolomics techniques to investigate soil metabolite characteristics in four different vegetation stages in karst regions: grassland (GL), brushwood (BW), secondary forest (SF) and primary forest (PF). We characterized changes in soil metabolite composition during karst vegetation succession, identified marker differential soil metabolites for each stage, and also determined the soil metabolite pathways as well as patterns of soil metabolite co-occurrence at different successional stages. We believe that quantification and characterization of soil compounds based on untargeted metabolomics will provide practical guidance for maintaining karst soil health.

## Results

### Composition and abundance of soil metabolites in the four karst vegetation types

**Figure 1:**
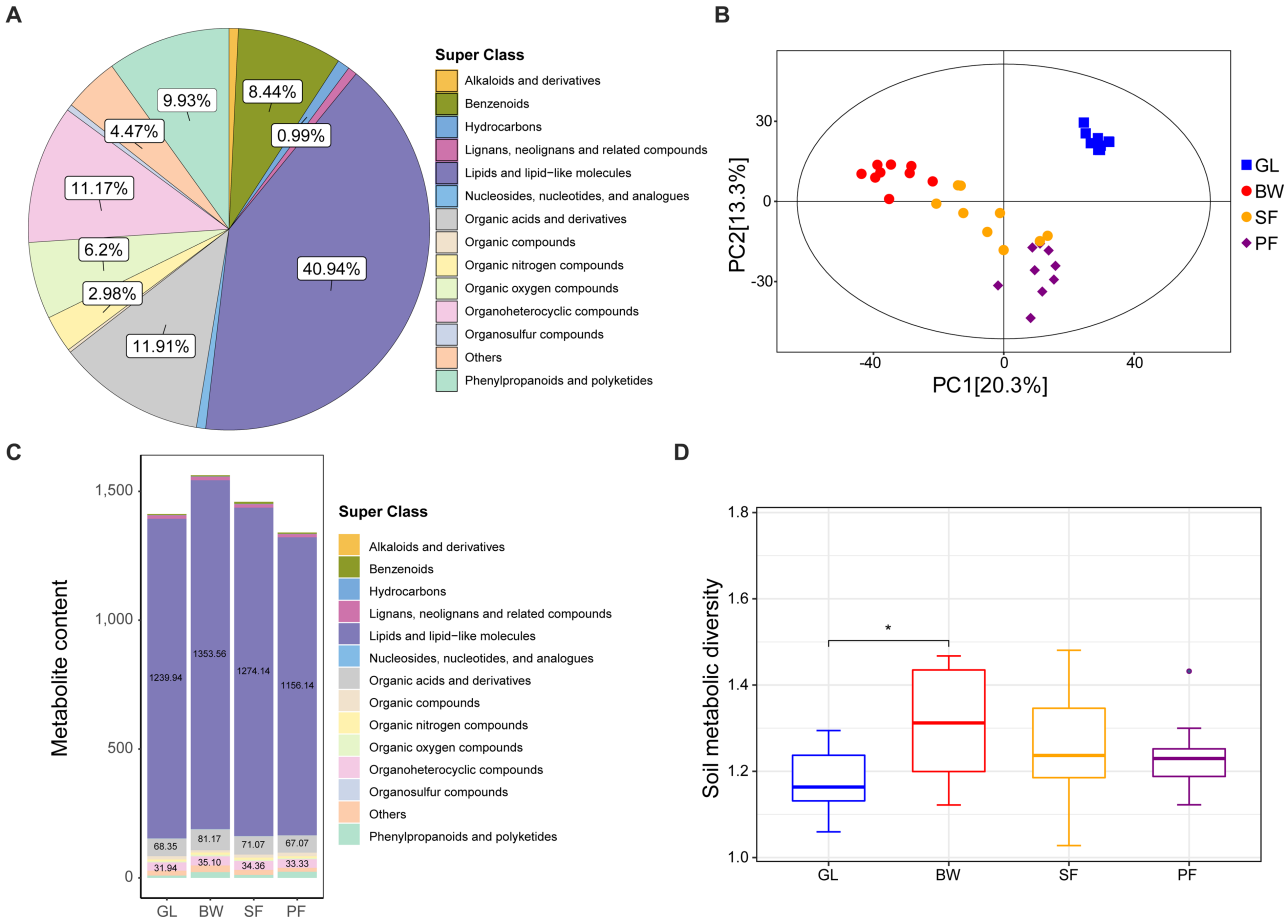
The metabolite identification and cluster analysis of GL, BW, SF, and PF groups. **(A)** Pie plot of identified metabolites. **(B)** PCA plots of metabolites identified from four groups. **(C)** Metabolite content under different stages of vegetation succession. **(D)** Soil metabolic diversity under different stages of vegetation succession. GL, grassland; BW, brushwood; SF, secondary forest; PF, primary forest.

We collected soil samples from GL, BW, SF and PF in the karst area of southern China (106°48′E, 22°31’N), with 9 samples from each group. The metabolite contents of karst soils from the four vegetation types were quantified using LC–MS/MS-based untargeted metabolomics. A total of 403 metabolites were detected and identified from all soil samples (Supplementary Table S1), with the majority falling into the categories of lipids and lipid-like molecules, organic acids and derivatives, organoheterocyclic compounds, phenylpropanoids and polyketides, benzenoids, organic oxygen compounds. Among these compounds, lipids and lipid-like molecules were the most abundant, accounting for 40.94% of the total metabolites, followed by organic acids and derivatives (11.91%), organoheterocyclic compounds (11.17%), and phenylpropanoids and polyketides (9.93%) (Figure 1A).

We conducted principal components analysis (PCA) to assess metabolite composition across the four vegetation stages. The distribution pattern of metabolite composition was vegetation-driven (Figure 1B), indicating that soil metabolomes were largely distinguishable across different karst vegetation types. Notably, some of the responsible metabolites overlapped in different vegetation soils, and their abundance varied across different vegetation soils. For example, BW had higher levels of lipids and lipid-like molecules, organic acids and derivatives, and organoheterocyclic compounds than the other groups (Figure 1C). In addition, GL and BW exhibited substantial variations in metabolite diversity, whereas SF and PF did not (Figure 1D).

### Differential expressed metabolites in four karst vegetation types

**Figure 2:**
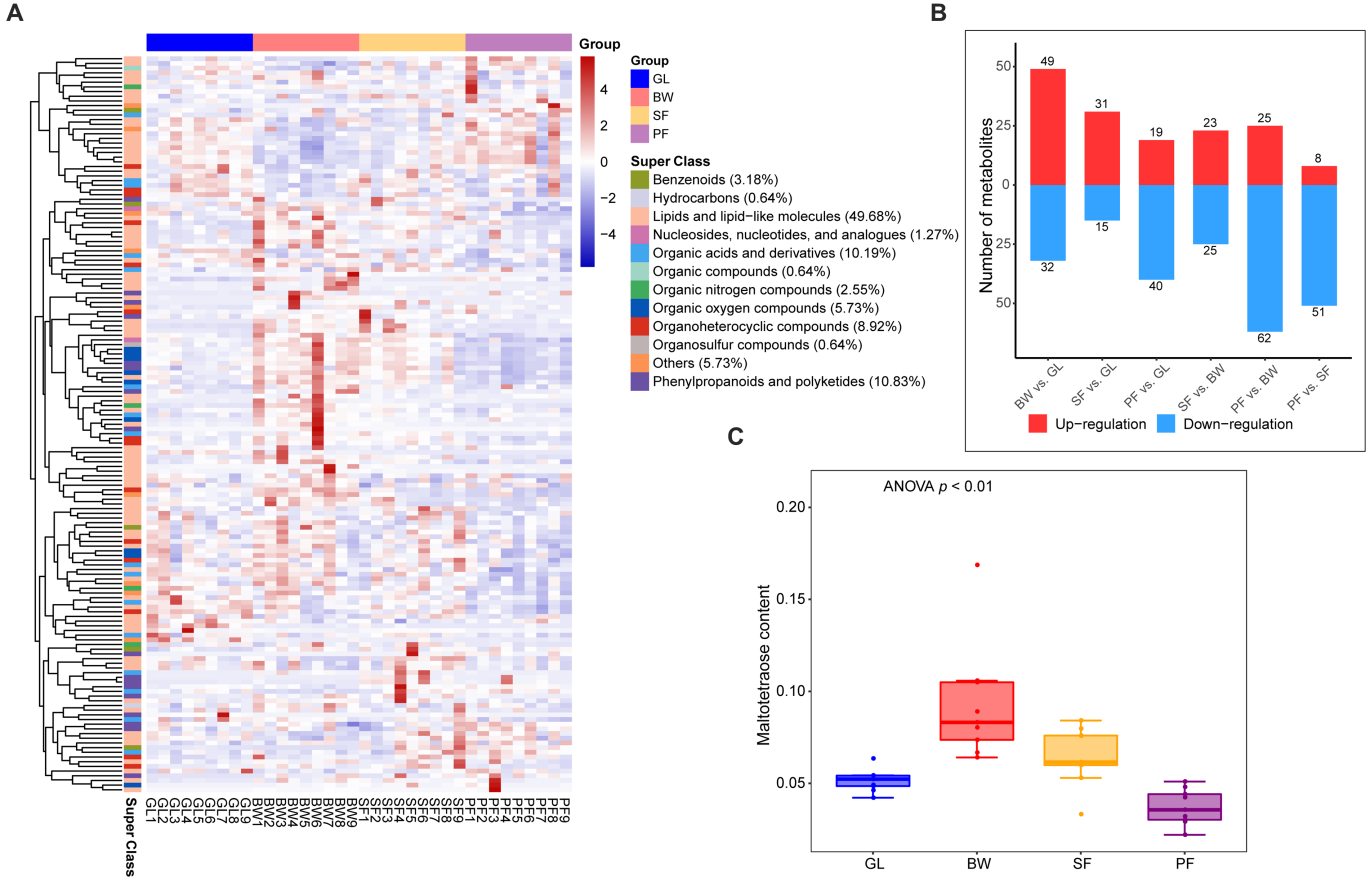
Differential metabolites screening and expression dynamics in soil under different stages of vegetation succession. **(A)** Cluster analysis between GL, BW, SF, and PF groups based on the DEMs. **(B)** The number of DEMs between the GL, BW, SF, and PF, red represents up-regulated metabolites, and blue represents down-regulated metabolites. **(C)** The content of maltotetraose under different stages of vegetation succession. GL, grassland; BW, brushwood; SF, secondary forest; PF, primary forest.

To further determine the soil metabolic variations among these four vegetation types, further screening for specific metabolites that were up-regulated or down-regulated between the four groups was conducted. The analysis with OPLS-DA model (VIP > 1, *p* < 0.05) revealed that 157 DEMs were significantly up-regulated or down-regulated among the vegetation types (Supplementary Table S2), mainly consisting of lipids and lipid-like molecules (49.68%), phenylpropanoids and polyketides (10.83%), and organic acids and derivatives (10.19%) (Figure 2A). Compared to the GL group, there were 81 DEMs (49 up-regulated and 32 down-regulated) in the BW group, 46 DEMs (31 up-regulated and 15 down-regulated) in the SF group, and 59 DEMs (19 up-regulated and 40 down-regulated) in the PF group (Figure 2B). Compared to the BW group, the SF group had 48 DEMs (23 up-regulated and 25 down-regulated), while the PF group had 87 DEMs (25 up-regulated and 62 down-regulated). There were 59 DEMs detected between the SF and PF groups, of which 51 were down-regulated in the PF group. We discovered considerable variations in soil metabolites from grassland to primary forest, suggesting that vegetation succession has a significant impact on the soil metabolism. Maltotetraose, a major product of starch metabolism by a variety of microorganisms in the soil, differed greatly in abundance among all groups, with the BW group having the greatest level (Figure 2C). In addition, we found 10 overlapped MEGs in BW vs. GL, SF vs. BW, PF vs. SF (Supplementary Figure S1), and these metabolites were altered at all stages of secondary succession and could serve as potential biomarkers. We also found differences in the abundance of 16 terpenoids, 8 flavonoids, and 6 isoflavonoids/neoflavonoids (Supplementary Figures S2–S4). These metabolic compounds are often secondary metabolites produced by plants, and discrepancies in their distribution in various vegetated soils may be due to vegetation type variations.

### Metabolic pathways of DEMs

**Figure 3:**
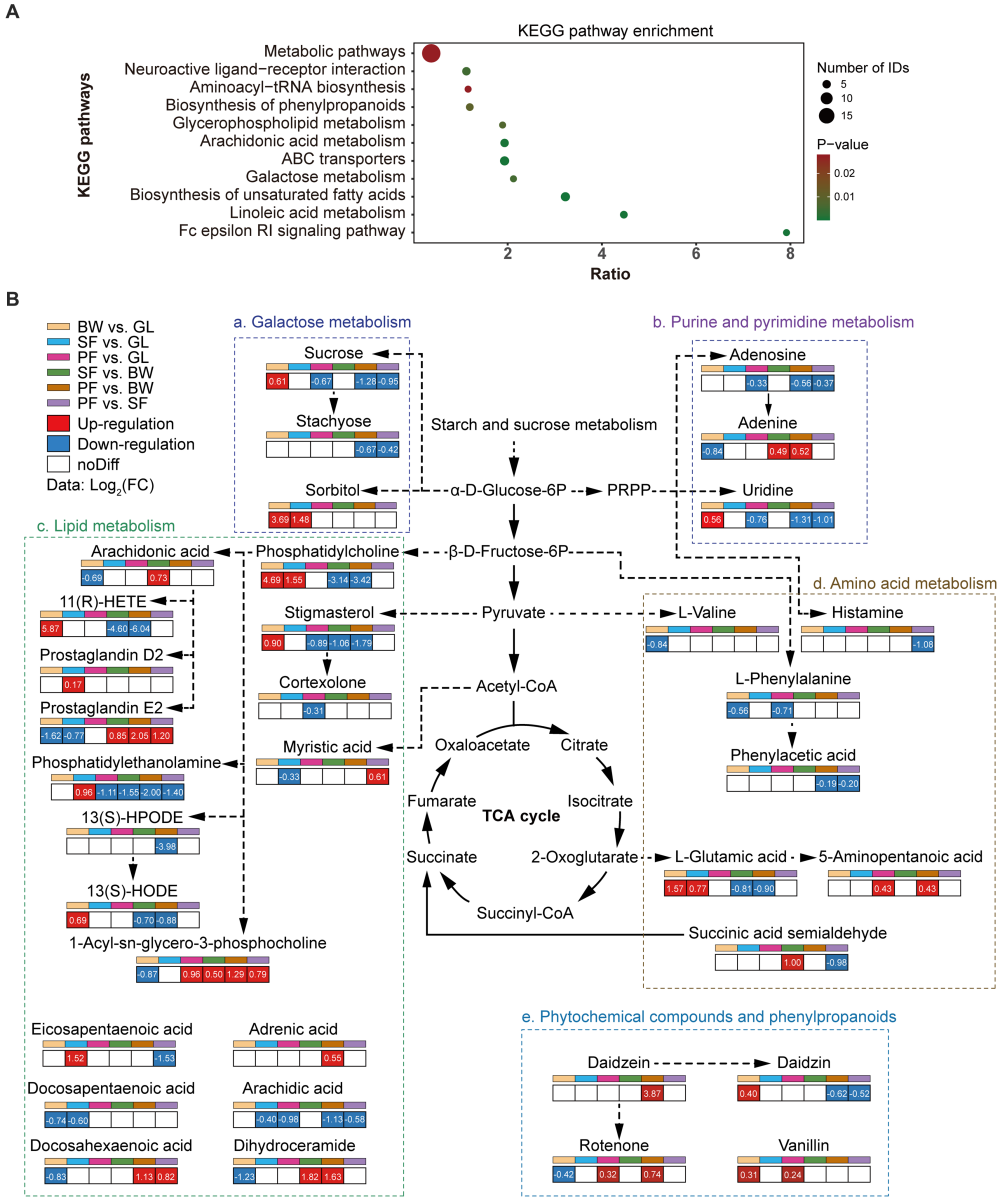
Dynamics of soil metabolic pathways of DEMs. **(A)** KEGG enrichment of differential metabolites. **(B)** The changes of soil metabolic pathways under different stages of vegetation succession. GL, grassland; BW, brushwood; SF, secondary forest; PF, primary forest.

To investigate the origin of DEMs and their potential effects on soil biological functions, pathway enrichment analysis was carried out with the differential metabolite KEGG ID. The enriched KEGG pathway revealed that showed that DEMs were mainly involved in the metabolic pathways, ABC transporters, biosynthesis of unsaturated fatty acids, arachidonic acid metabolism, and neuroactive ligand-receptor interaction (Figure 3A). The enrichment of DEMs in basal metabolic pathways suggested that different vegetation types altered the basic metabolic network of soil. The primary metabolic networks (carbohydrates, amino acids and lipids) of soil differed significantly across vegetation types (Figure 3B). The expression of the metabolic pathways for galactose metabolism as well as purine and pyrimidine metabolism were considerably reduced in the PF group: sucrose (−1.28 to −0.67), stachyose (−0.67 to −0.42), adenosine (−0.56 to −0.33) and uridine (−1.31 to −0.76). In comparison to the GL group, sucrose was upregulated in the BW group (0.61 times), and sorbitol was also upregulated in both BW and SF groups (3.69 times and 1.48 times, respectively). The SF and PF groups had higher levels of adenine than the BW group, by 0.49 and 0.52 times, respectively. The lipid metabolism pathways showed that 1-Acyl-sn-glycero-3-phosphocholine metabolism (0.79 to 1.29) accumulated in large quantities in the PF group, while phosphatidylethanolamine (−2.00 to −1.11) and arachidic acid (−1.13 to −0.58) were decreased. Compared with other stages of vegetation succession, the BW group had considerably higher levels of phosphatidylcholine, 11(R)-HETE, 13(S)-HODE, stigmasterol, whereas 1-Acyl-sn-glycero-3-phosphocholine and dihydroceramide levels were lower. Compared to other groups, amino acid synthesis and metabolism were inhibited in the PF group, specifically in histamine (−1.08 times vs. SF), L-phenylalanine (−0.71 times vs. GL), phenylacetic acid (−0.19 times vs. BW and − 0.20 times vs. SF), L-glutamic acid (−0.90 times vs. BW), and succinic acid semialdehyde (−0.98 times vs. SF). Daidzein and rotenone are involved in isoflavone biosynthesis, and their expression was up-regulated in PF compared to SF.

### Metabolite-metabolite correlation analysis

**Figure 4:**
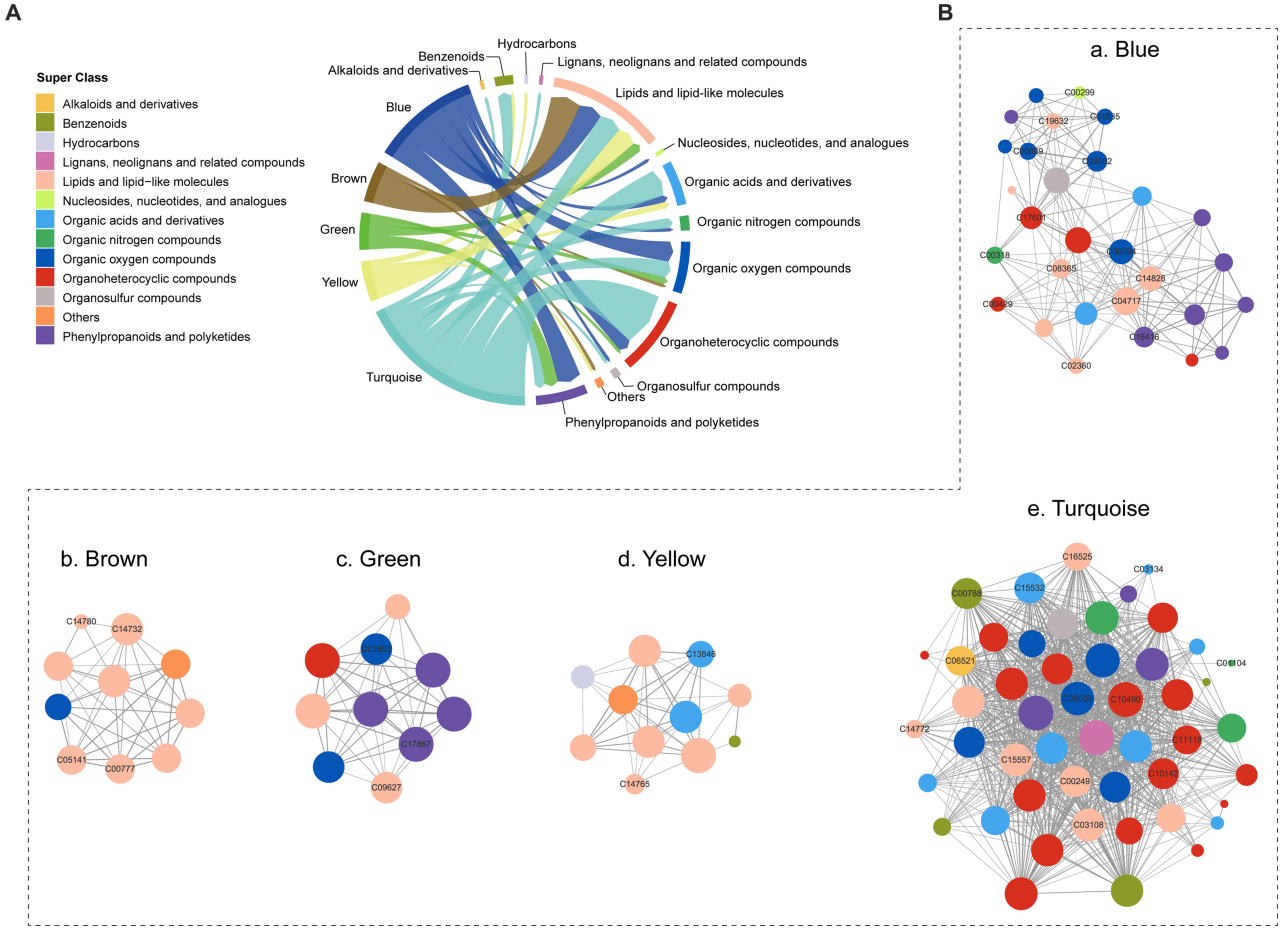
Co-occurrence network of the metabolites. **(A)** The composition of co-occurrence metabolite modules. **(B)** Co-occurrence network of five modules.

A co-occurrence network was constructed using WGCNA to analyze the metabolic interactions and dependencies of 403 metabolites. As a result, five co-occurrence metabolite modules were identified (Supplementary Table S3), with metabolites primarily originating from lipids and lipid-like molecules, organoheterocyclic compounds, organic oxygen compounds, and phenylpropanoids and polyketides, and organic acids and derivatives (Figure 4A). In the blue module, some nodes (C04717, C14828) involved in linoleic acid metabolism exhibited higher connectivity than others. The brown and yellow modules were mainly composed of lipids and lipid-like molecules, but the turquoise module contained a broader range of metabolites, including organoheterocyclic compounds, organic acids and derivatives and lipids and lipid-like molecules (Figure 4B).

**Figure 5:**
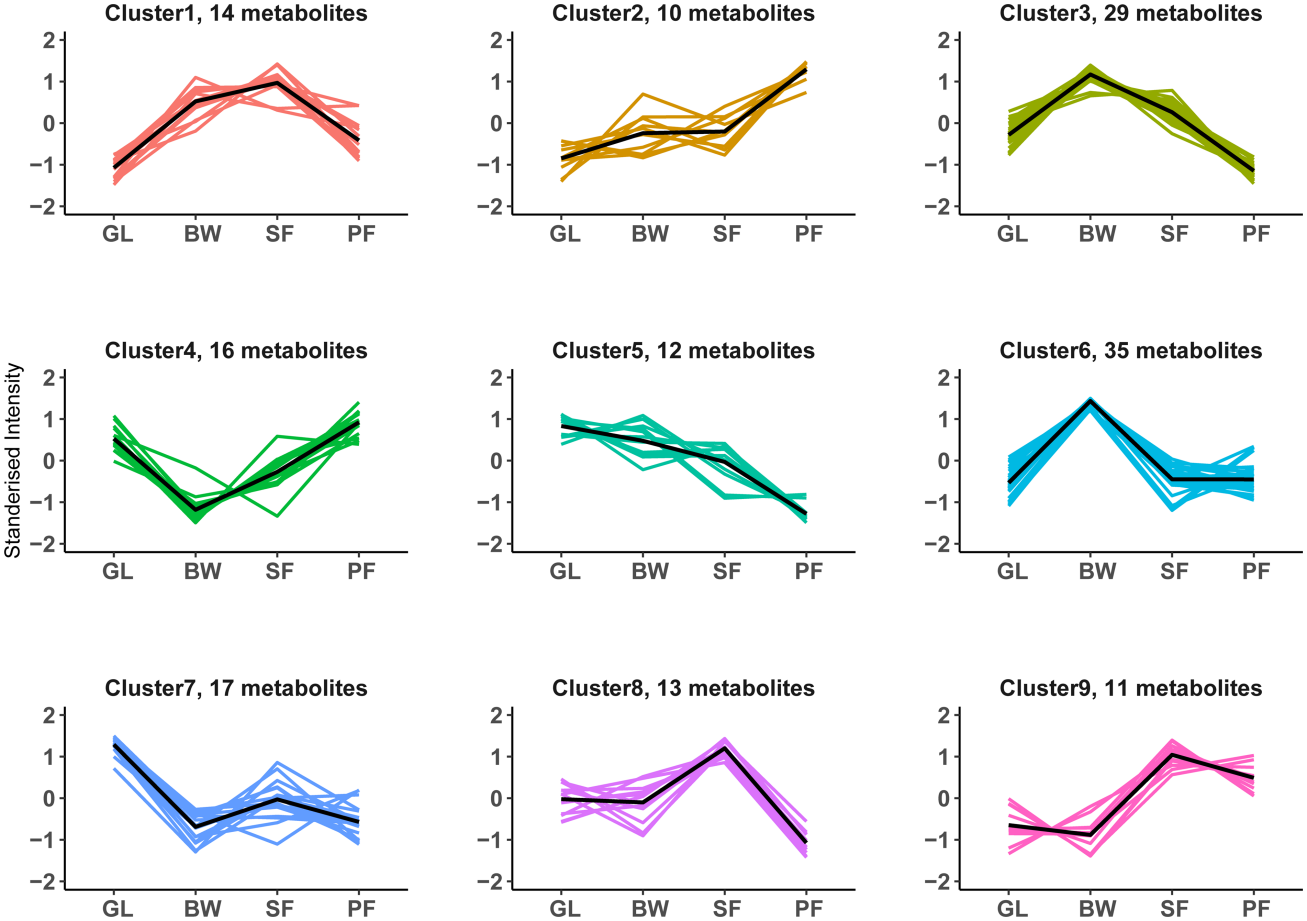
K-means clustering patterns of DEMs along vegetation succession. All 157 DEMs were clustered into 9 groups using K-means clustering. The black line shows average expression z-scores to visualize the dominant expression trend of each cluster. Each line in the figure represents an expression value of the corresponding DEMs. GL, grassland; BW, brushwood; SF, secondary forest; PF, primary forest.

To investigate the composition of soil metabolites with similar expression patterns across vegetation types, we performed a K-means cluster analysis of the DEMs. A total of 157 DEMs were successfully divided into 9 clusters (designated as clusters 1–9), each of which had its own distinct expression pattern that peaked across vegetation types (Figure 5; Supplementary Table S4). Metabolites within a cluster had similar expression patterns across successional stages. Cluster 2 (*n* = 10) described an increase with secondary succession, whereas cluster 5 (*n* = 12) featured a reduction with secondary succession. Metabolites in clusters 3 (*n* = 29) and 6 (*n* = 35) had distinctly higher content at the BW stage, whereas those in clusters 4 (*n* = 16) and 7 (*n* = 17) had the lowest at the BW stage. Metabolites in clusters 1 (*n* = 14), 8 (*n* = 13), and 9 (*n* = 11) exhibited significantly higher content at the SF stage.

## Discussion

### The composition of metabolites in different vegetation soils of karst

In this study, we compared the differences in soil metabolite composition among different vegetation stages in karst areas and revealed the expression patterns of different metabolites with vegetation succession. Plants and soil microbes modulate their metabolic activities as karst vegetation succession occurs, changing the soil metabolic spectrum (Zhu et al., 2012; Hu et al., 2017). Significant variations in numerous metabolites were observed as secondary plant succession progressed, demonstrating that karst vegetation succession dramatically affected the soil metabolic spectrum (Figures 2, 3). Among all identified metabolites, 157 differential metabolites were observed in response to successional processes (Supplementary Table S2). There were 81 metabolites with significant differences in abundance and a significant increase in metabolite diversity from GL to BW stage (Figure 1D). Due to the harsher environmental conditions in grasslands, plants may secrete antioxidant substances, protective proteins and resilient enzymes in response to harsh environmental stresses. In the current study, five metabolites were up-regulated in GL compared to BW, SF, and PF: dihydro-4-mercapto-3(2H)-furanone, piperidine, cycloartenol, trans-3-feruloylcorosolic acid, and ethiin (Supplementary Table S2), with cycloartenol being a key precursor for the biosynthesis of antioxidant substances in plants (Zhang et al., 2017). Compared to the GL, the BW is able to use light and nutrient resources more efficiently (Zhu et al., 2012). From GL to BW, the soil metabolite changed significantly, accumulating more sugars and secondary metabolites including bifurcose and ursolic acid.

### Dynamics of key metabolites with karst vegetation succession

We identified 10 karst soil metabolites that changed dynamically throughout the vegetation succession process (Supplementary Figure S1). Bifurcose, maltopentaose, and maltotetraose are sugars that microbes can use as carbon sources and to generate energy for their usual metabolism. There was an increase in the content of these sugars in the brushwood stage and then a decrease at the SF and PF stages. The metabolites secreted by the plants into the soil also changed significantly with the vegetation succession. Sterebin E, a diterpenoid synthesized by plants, decreased at the BW stage, increased at the SF stage, and finally declined at the PF stage. Terpenoids such as diterpenoids and terpenoids, as important substances secreted by plants into the soil, can not only influence the chemical properties of the soil, but also the growth and metabolism of soil microorganisms (Huang and Osbourn, 2019). From the GL to BW stage, there was a significant increase in triterpenoids such as soyasapogenol E and ursolic acid, as well as a significant increase in diterpenoids such as phytocassane D, and sesquiterpenoids such as capsidiol, solavetivone, and 1,4,9-Cadinatriene (Supplementary Figure S2). Soil flavonoids and isoflavonoids are also involved in responding to plant adversity stress and improving plant growth adaptation (Dixon and Steele, 1999). Differential expression of eight flavonoid metabolites was detected during secondary karst succession, with 3′,4′,5′,7,8-Pentamethoxyflavan, hexamethylquercetagetin, and sakuranetin first increasing at the BW and then decreasing at SF (Supplementary Figure S3). Isoflavonoids and neoflavonoids, such as daidzin and mammeigin, also increased significantly at the BW stage (Supplementary Figure S4).

### Metabolic pathway and interaction effect of DEMs

KEGG metabolic pathway analysis showed that the soil differential metabolites were enriched in metabolic pathways such as the biosynthesis of unsaturated fatty acids, and each key pathway was mostly shared by more than one metabolite. Arachidonic acid, arachidic acid, eicosapentaenoic acid, docosahexaenoic acid, docosapentaenoic acid, and adrenic acid were all involved in the biosynthesis of unsaturated fatty acids and were significantly differentially expressed at different successional stages. Among them, arachidonic acid, docosapentaenoic acid and docosahexaenoic acid were metabolized at reduced levels from the GL to the BW stage (Figure 3B). Some previous studies had found that changes in relative abundance of fatty acids are often attributed to shifts in microbial community compositions (Bossio and Scow, 1998; Iyyemperumal and Shi, 2007). The lipid constitute of microbial membrane was shifted by environmental changes (He et al., 2023). We did notice a large change in fatty acids throughout secondary karst succession, implying that alterations in cell membrane composition may be a crucial distinction between microorganisms at different vegetation stages. Sucrose, stachyose and sorbitol are involved in the galactose metabolism, a microbially important process (Shi et al., 2023). Sucrose and sorbitol increased during the succession from GL to BW, while sucrose and stachyose decreased during the succession from SF to PF (Figure 3B). In karst soils, some metabolites become major factors in the network, and these metabolites strongly co-occur during the secondary succession of vegetation, e.g., 5-KETE, all-trans-Retinoic acid, estriol. In K-means clustering, cluster 3 displayed more specific terms involved in galactose metabolism and carbohydrate metabolism compared to the other clusters (Supplementary Table S4). Sucrose, stachyose, bifurcose, maltopentaose and maltotetraose in cluster 3 were carbon compounds that have been shown to be carbon sources for soil microorganisms (Gunina and Kuzyakov, 2015). During plant growth, litter or root exudates alter the concentration of these sugars in the soil (Sokol et al., 2019; Fan et al., 2022), which may affect microbial activity. The biosynthesis of unsaturated fatty acids was enriched by DEMs in clusters 4, 5, 6, and 7. However, the expression patterns of these four clusters are different. Adrenic acid and docosahexaenoic acid in cluster 4, and arachidonic acid and docosapentaenoic acid in cluster 7, decreased at BW stage, and then increased at SF stage, while cluster 7 experienced another decrease at PF stage.

### Effects of environmental factors on soil metabolism

Karst vegetation succession alters soil qualities, particularly its physical and chemical properties. It has been shown that with the degradation of karst vegetation, the expression of amino acid metabolism genes in soil microorganisms decreases, resulting in changes in the activity of related enzymes (Li et al., 2021). In the current study, amino acid metabolism was found to change with vegetation succession, possibly as a result of changes in soil enzyme activities. Vegetation restoration has been found to increase organic matter accumulation and improve soil moisture conditions in karst ecosystems (Liu et al., 2015; Xiao et al., 2018). Many studies have identified an increasing trend in soil organic carbon, nitrogen content and soil moisture and a decreasing trend in pH from grassland to forest (Liu et al., 2015; Xue et al., 2017; Xiao et al., 2018). These changes in soil properties enable microbes to respond to their immediate environmental conditions, and thus indirectly influence metabolite pools (Wang et al., 2021; Tang et al., 2024). However, our current study did not explore the interaction of soil metabolism with microbes and soil properties. More research is needed to understand the interaction patterns of soil metabolites, soil properties and microbial communities, as well as their substantial impacts on karst ecosystems.

## Conclusion

This study used untargeted metabolomics techniques for the identification and characterization of molecules in soils, revealing the effects of karst vegetation succession on soil metabolism. The results demonstrated that karst vegetation succession dramatically impacts soil metabolic processes. The significantly changed metabolites along succession were involved in lipids and lipid-like molecules, phenylpropanoids and polyketides, organic acids and derivatives. The diversity index of metabolites significantly differed in grassland and brushwood. The functional pathways of the changed soil metabolite were found to be more involved in galactose, lipids and amino acids metabolism. Our results highlight the soil metabolite spectrum in different karst vegetation types, which will be useful for future restoration and conservation of local karst soil resources.

## Materials and methods

### Study area

The study area is located in Longzhou County, Chongzuo City, Guangxi Province (106°48′E, 22°31’N), which is a typical karst landform. It belongs to the subtropical humid monsoon climate type. The unique topographic features and hydrological processes cause weak structural stability of the ecosystem, forming a typical ecologically fragile karst area susceptible to disturbance and with poor resilience. Nine replicated plots of in each vegetation type (GL, BW, SF, and PF) were established. The dominant plant species in GL include *Alocasia odora*, *Bidens pilosa*, and *Pueraria montana* var. *lobata*; the dominant plant species in the BW are *Cipadessa baccifera* and *Alchornea trewioides*; in SF, the dominant plant species include *Vitex kwangsiensis Streblus tonkinensis*, *Cleistanthus sumatranus*, and *Ficus hispida*; while in PF, the dominant plant species are *Excentrodendron tonkinense*, *Deutzianthus tonkinensis*, and *Litsea variabilis* var. *oblonga*.

### Sample collection

Soil samples in each plot were collected from the surface soils (0–10 cm), covering GL (9 samples), BW (9 samples), SF (9 samples) and PF (9 samples) in April of 2022. Five soil cores were selected in a diagonal pattern from each plot using a soil corer and mixed to form one representative composite sample. All soil samples were then passed through a 2 mm sieve to remove visible roots and stones. Each soil sample was transferred to a 50 mL sterile centrifuge tube and placed in a dry ice tank. The samples were transported to the laboratory, and the sterile centrifuge tube was immediately stored in an ultra-low temperature refrigerator at −80°C for subsequent extraction of soil metabolites.

### Metabolite extraction

Metabolite extractions were conducted following the methods reported in a previous study (Alseekh et al., 2021). For extraction, 100 mg of sample was weighted to an EP tube, and 1,000 μL extraction solution (methanol: water = 3: 1, with isotopically-labeled internal standard mixture) was added, then homogenized at 35 Hz for 4 min and sonicated in an ice-water bath for 5 min. The homogenization and sonication cycle were repeated for 3 times. The samples were incubated at −40°C for 1 h and then centrifuged at 12000 rpm (RCF = 13,800(×g), R = 8.6 cm) for 15 min at 4°C. The resulting supernatant was transferred to a fresh glass vial for analysis. The quality control (QC) sample was prepared by mixing an equal aliquot of the supernatants from all of the samples.

### LC–MS/MS analysis

Sample extracts were analyzed by LC–MS/MS (Xiao et al., 2012) using an UHPLC system (Vanquish, Thermo Fisher Scientific) with a UPLC HSS T3 column (2.1 mm × 100 mm, 1.8 μm) coupled to Orbitrap Exploris 120 mass spectrometer (Orbitrap MS, Thermo) (Dunn et al., 2011). The mobile phase consisted of 5 mmol/L ammonium acetate and 5 mmol/L acetic acid in water (A) and acetonitrile (B). The auto-sampler temperature was 4°C and the injection volume was 2 μL.

The Orbitrap Exploris 120 mass spectrometer was used for its ability to acquire MS/MS spectra on information-dependent acquisition (IDA) mode in the control of the acquisition software (Xcalibur, Thermo). In this mode, the acquisition software continuously evaluates the full scan MS spectrum. The ESI source conditions were set as follows: sheath gas flow rate as 50 Arb, Aux gas flow rate as 15 Arb, capillary temperature 320°C, full MS resolution as 60,000, MS/MS resolution as 15,000 collision energy as 10/30/60 in NCE mode, spray Voltage as 3.8 kV (positive) or − 3.4 kV (negative), respectively.

### MS data and statistical analyses

Raw data were converted to mzXML format using ProteoWizard and processed with an in-house program, which was developed using R and based on XCMS, for peak detection, extraction, alignment, and integration. An in-house MS2 database was then used for metabolite annotation. The cutoff for annotation was set to 0.3.

Principal component analysis (PCA) was performed on pairs of samples using SIMCA software (V16.0.2) to determine the overall distribution trends among the samples. The data were formatted for logarithmic (LOG) transformation and centralization (CTR), and then automatically modeled. Orthogonal projections to latent structures-discriminant analysis (OPLS-DA) (Reinke et al., 2018) analysis was conducted by SIMCA software (V16.0.2) to identify the DEMs between different groups. Metabolites with multivariate and univariate statistical significance (VIP>1.0 and *p* < 0.05) for Student’s *t*-test were identified as DEMs between two groups. Clustering of the DEMs between different successional stages was analyzed using K-means.

The Kyoto Encyclopedia of Genes and Genomics (KEGG) database (Kanehisa et al., 2015) was utilized to conduct KEGG pathway analysis. Pathway enrichment analysis was performed using MBROLE 2.0 (López-Ibáñez et al., 2016), and pathways with *p*-values < 0.05 were considered significantly enriched. The metabolite co-occurrence network was constructed using the WGCNA R package (Langfelder and Horvath, 2008). Cytoscape 3.10.0 software (Otasek et al., 2019) was used to visualize the network of metabolites.

## Data availability statement

The data presented in the study are deposited in the MetaboLights repository, accession number MTBLS10439.

## Author contributions

CZ: Writing – original draft, Writing – review & editing. CH: Formal analysis, Writing – review & editing. CX: Methodology, Writing – original draft. ZZ: Project administration, Validation, Writing – review & editing. GH: Project administration, Writing – review & editing.
