# Supplementary material for: Metabolomics reveals changes in soil metabolic profiles during vegetation succession in karst area
Source: Front Microbiol. 2024 Jun 26;15:1337672. doi: 10.3389/fmicb.2024.1337672 (PMC11233535; doi:10.3389/fmicb.2024.1337672)
Supplement: Supplementary file 1 [file Presentation_1.pdf]

Supplementary figures for "Metabolomics reveals changes in soil metabolic profiles during vegetation succession in karst area"

Chaofang Zhong[1], Cong Hu[1], Chaohao Xu[1], Zhonghua Zhang[1,*], Gang Hu[1,*]

[1] Key Laboratory of Wildlife Evolution and Conservation in Mountain Ecosystem of Guangxi, College of Environmental and Life Sciences, Nanning Normal University, Nanning 530001, China

[*] Corresponding authors. E-mail: gxtczzh@126.com, ahhugang@126.com

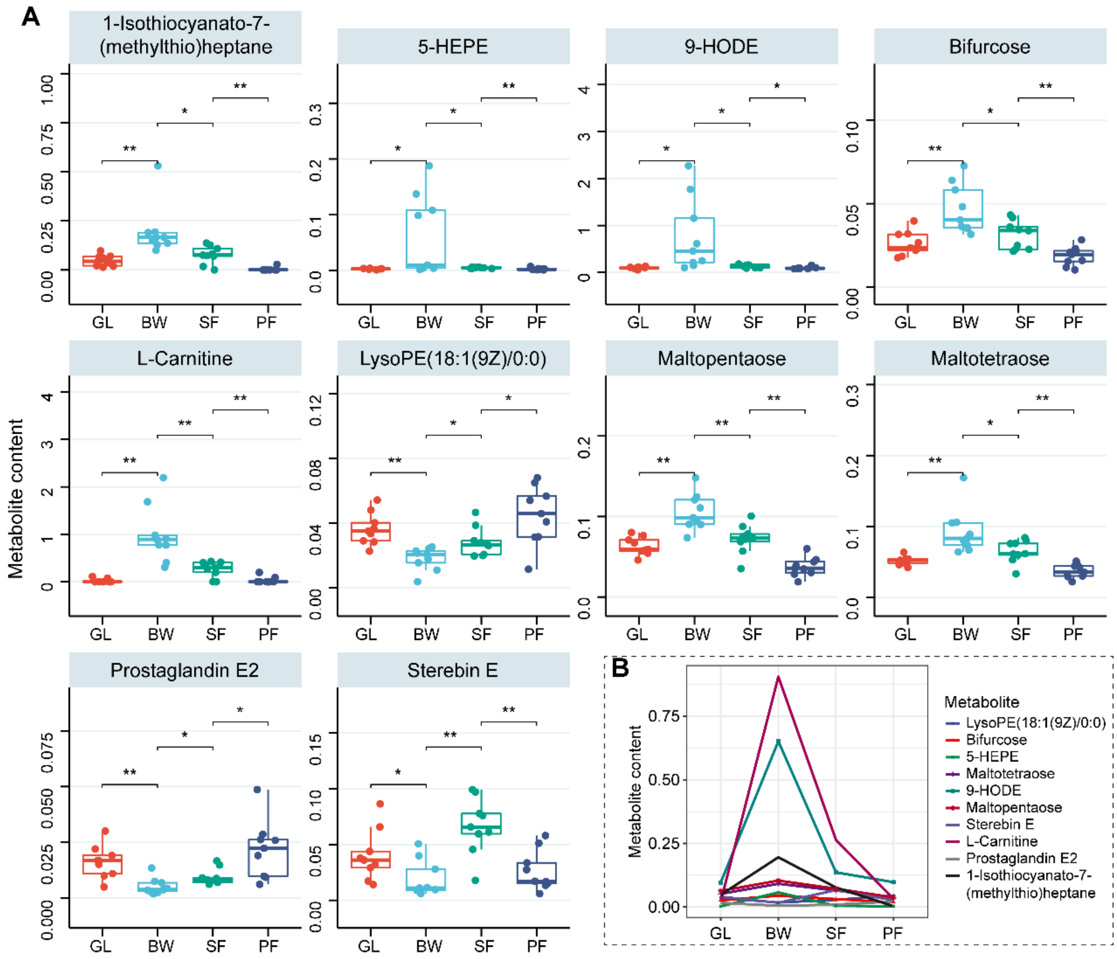

Supplemental Figure S1. Dynamics of 10 overlap metabolites in BW vs. GL, SF vs. BW, PF vs. SF. (GL = grassland, BW = brushwood, SF = secondary forest, and PF = primary forest).

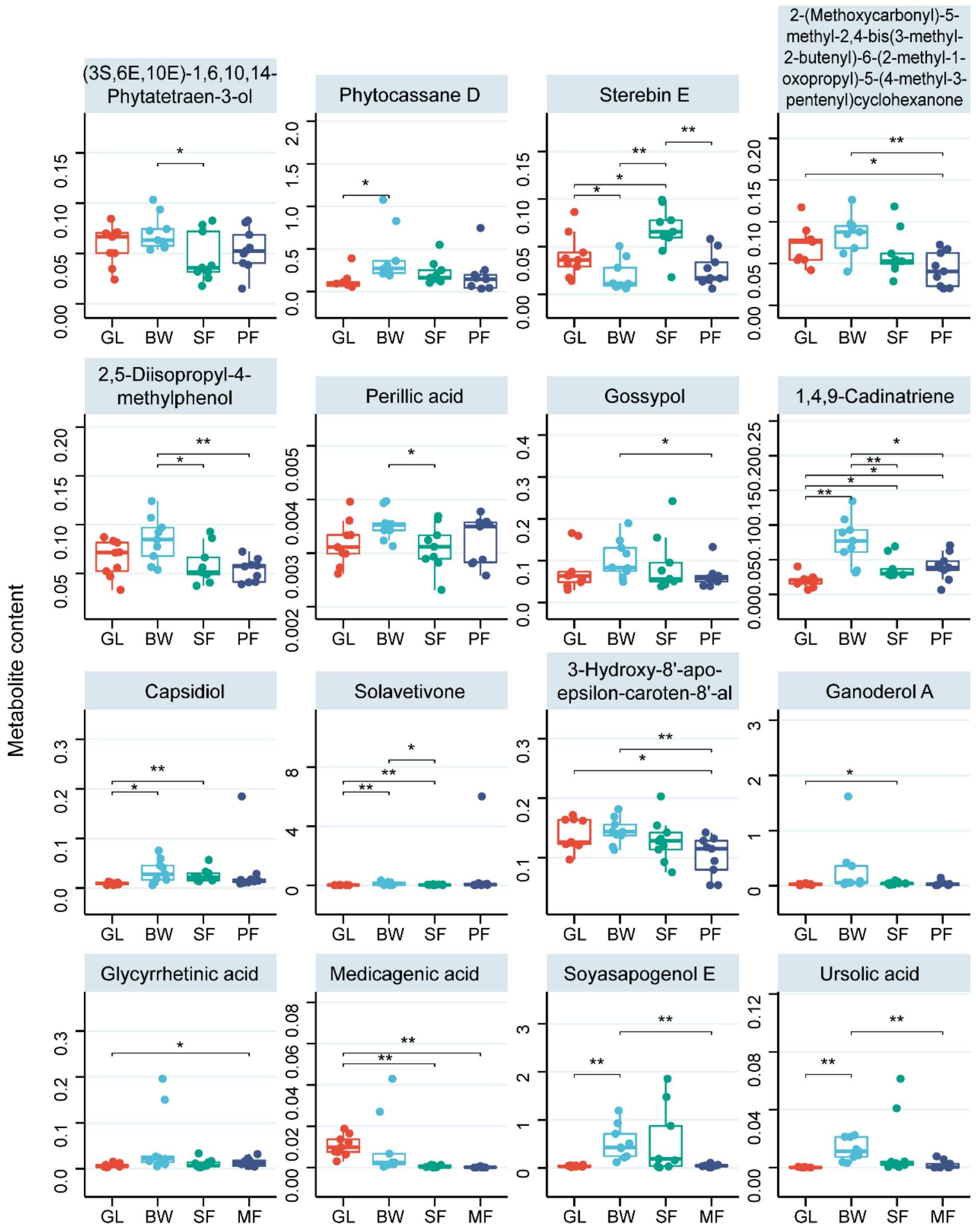

Supplemental Figure S2. Differentially expressed terpenoids under different stages of vegetation succession. (GL = grassland, BW = brushwood, SF = secondary forest, and PF = primary forest).

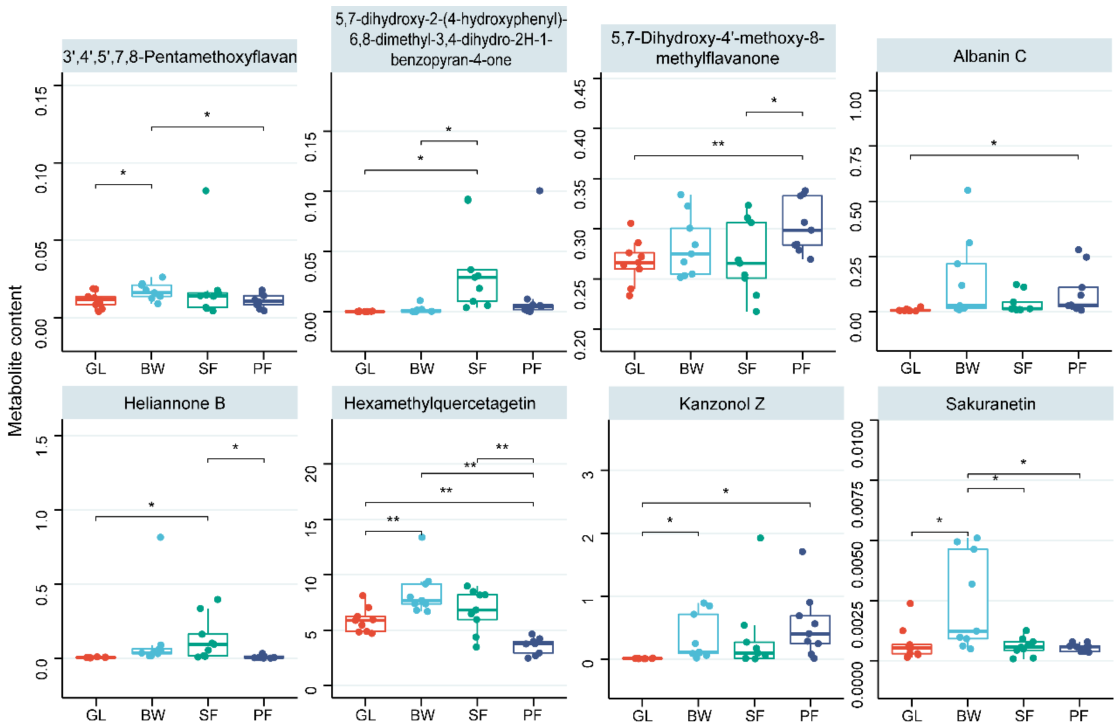

Supplemental Figure S3. Differentially expressed flavonoids under different stages of vegetation succession. (GL = grassland, BW = brushwood, SF = secondary forest, and PF = primary forest).

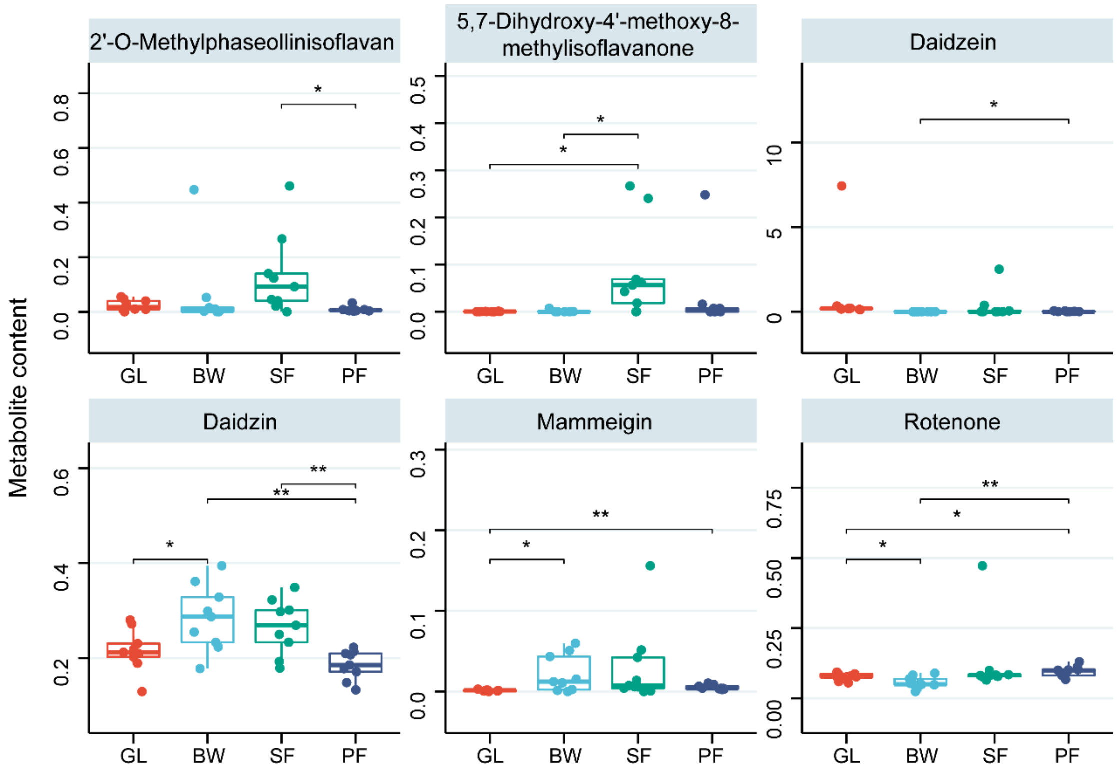

Supplemental Figure S4. Differentially expressed isoflavonoids/neoflavonoids under different stages of vegetation succession. (GL = grassland, BW = brushwood, SF = secondary forest, and PF = primary forest).
